# Is There an Ace Up One's Sleeve in the Preanalytical Phase of the Circulating Tumor DNA Analysis?

**DOI:** 10.1002/biof.70095

**Published:** 2026-03-29

**Authors:** Juscelino Carvalho de Azevedo Junior, Douglas Rafael da Cruz Carneiro, Fernanda Jardim da Silva, Ana Beatriz Lima Belicha, Anna Carolina Lima Rodrigues, Williams Fernandes Barra, Alessandro Leal, Danielle Queiroz Calcagno

**Affiliations:** ^1^ Núcleo de Pesquisas em Oncologia Universidade Federal do Pará Belém Pará Brazil; ^2^ Programa de Residência Multiprofissional em Saúde (Oncologia), Hospital Universitário João de Barros Barreto Universidade Federal do Pará Belém Pará Brazil; ^3^ Instituto de Ciências da Saúde Universidade Federal do Pará Belém Pará Brazil; ^4^ The Sidney Kimmel Comprehensive Cancer Center Johns Hopkins University School of Medicine Baltimore Maryland USA

**Keywords:** cell death, liquid biopsy, preanalytical variables, precision medicine, standardization of preanalytical methods, tumor biomarkers

## Abstract

Circulating tumor DNA (ctDNA) analysis has emerged as a pivotal minimally invasive tool for early detection, monitoring, and treatment stratification in cancer patients. However, the accuracy and reliability of ctDNA assays are profoundly influenced by preanalytical variables. This review discusses the impact of biological features (circadian rhythm, age, and sex), lifestyle factors (diet, smoking, and physical activity), as well as technical aspects such as hemolysis, leukocyte lysis, and delayed plasma separation on ctDNA integrity and concentration. Fluctuations in ctDNA levels driven by these factors highlight the need for clear guidelines regarding precollection timing, dietary restrictions, and sample processing. Furthermore, the adoption of harmonized protocols is essential to reduce variability and improve reproducibility across clinical and research settings.

## Graphical abstract

Preanalytical biological, lifestyle, and technical factors can substantially increase background cfDNA, masking tumor‐derived ctDNA. This review emphasizes the need for careful control and standardization of these variables to improve ctDNA detection, analytical sensitivity, and clinical interpretation in oncology.

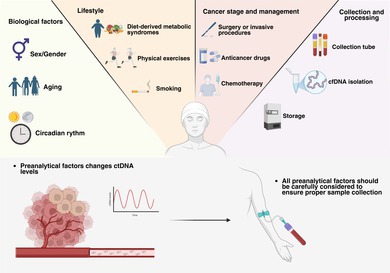

## 1 Background

Cancer is a complex and multistage disease with high morbidity and mortality worldwide [1, 2]. Current diagnostic and prognostic approaches are insufficient for effective clinical management of the disease. Tumor markers, imaging tests, and tissue biopsy cannot characterize the complex temporal or spatial heterogeneity of tumors and metastatic lesions [1, 2, 3, 4, 5].

Liquid biopsy (LB) has emerged as a minimally invasive method that offers numerous advantages, including ease of repeat sampling, reduced patient discomfort, and the ability to provide real‐time insights into tumor dynamics and treatment efficacy [6, 7, 8]. Specifically, circulating tumor DNA (ctDNA) has critical information for early cancer detection, monitoring disease progression, evaluating treatment response, and identifying potential resistance mechanisms [6].

ctDNA is a subtype of cell‐free DNA (cfDNA) present in body fluids, mainly blood, that consists of DNA fragments derived from cancer cell death or microvesicle active release [9]. These fragments contain tumor‐specific alterations and reflect molecular alterations, including mutations, copy number variations, chromosomal rearrangements, and methylation changes [10]. Importantly, cfDNA in circulation originates predominantly from nonneoplastic tissues, with ctDNA typically representing approximately 0.1%–1.0% of the total cfDNA pool. Consequently, fluctuations in total cfDNA levels do not necessarily reflect proportional changes in ctDNA burden [11].

**FIGURE 1:**
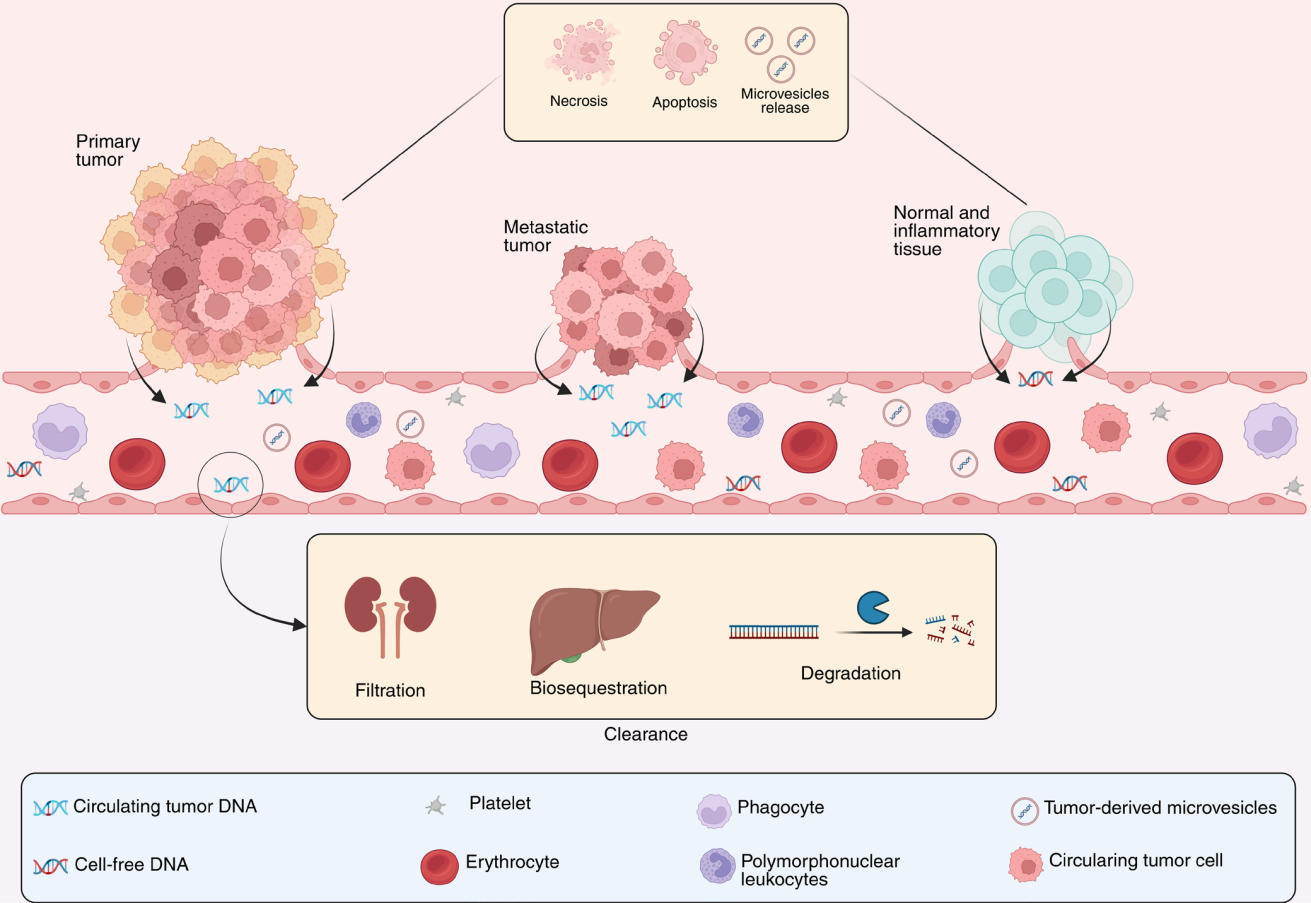
Biological aspects of cfDNA and ctDNA. Normal and inflammatory tissues, as well as primary and metastatic tumors, release cfDNA and ctDNA into the bloodstream primarily through apoptosis, necrosis, and the active release of microvesicles. These DNA fragments are cleared from circulation via glomerular filtration, biosequestration by the reticuloendothelial system, and degradation by serum nucleases.

In healthy individuals, cfDNA levels in the bloodstream vary between 0 and 100 ng/mL, whereas in cancer patients vary between 0 and 1000 ng/mL according to tumor type, size, and stage [8]. Following release by normal or malignant cells, cfDNA can be cleared by the reticuloendothelial system, kidneys, and serum nucleases (Figure 1) [11, 12, 13].

Since ctDNA represents only a small fraction of cfDNA and often exhibits a shorter half‐life, its analysis requires high‐sensitivity and specificity methods. These include next‐generation sequencing (NGS), quantitative real‐time polymerase chain reaction (qPCR), and droplet digital PCR (ddPCR). Despite major technical progress in LB workflows, the standardization of preanalytical procedures remains inadequate [14, 15].

These procedures encompass both pre‐preanalytical factors, occurring before blood collection (such as biological characteristics and lifestyle‐related variables), and preanalytical factors after collection, including sample handling, processing, storage, and DNA extraction. Variability at any of these stages can substantially influence cfDNA concentration, ctDNA fraction, and analytical sensitivity [16].

Notably, several biological and lifestyle‐related factors may increase background cfDNA without a concomitant rise in tumor‐derived ctDNA, leading to a dilution effect that reduces the ctDNA and increases the risk of false‐negative results. Therefore, lack of control over preanalytical variability can result in significant discrepancies in ctDNA analysis, ultimately affecting clinical interpretation and decision‐making [16].

The present review discusses the pre‐preanalytical factors, emphasizing the significant impact of biological features (circadian rhythm, age, and sex) and lifestyle factors (diet, smoking, and physical exercises) at the levels of cfDNA and ctDNA. The present manuscript does not aim to review cfDNA‐based applications per se, but rather to highlight how variations in total cfDNA influence ctDNA fraction, detectability, and analytical performance specifically in the oncology context. We also discuss the collection and processing methods on ctDNA integrity and concentration. Addressing these issues is critical for ensuring the accuracy and reliability of ctDNA‐based diagnostics and monitoring in oncology.

## 2 Could the ctDNA Analysis Be Influenced by the Circadian Rhythm?

The circadian rhythm consists of complex neural and molecular pathways that control the sleep–wake cycle, which is essential for human health. Night darkness triggers the retina to activate the suprachiasmatic nucleus from the hypothalamus, prompting the pineal gland to release melatonin. This hormone, combined with oxytocin and vasopressin, induces sleepiness, marking the rest phase. Conversely, daylight suppresses melatonin production, fostering wakefulness or an active state [17, 18].

The regulatory process of the circadian rhythm involves the activation of the period (*PER*) and cryptochrome (*CRY*) genes via BMAL1/CLOCK heterodimeric complexes that bind to E/E′‐boxes on DNA during the rest phase. Subsequently, the accumulation of PER/CRY heterodimers inhibits *BMAL1/CLOCK* transcription. The degradation of the PER and CRY proteins reactivates the transcription cycle of these genes, establishing a transcriptional feedback loop that sustains the circadian rhythm [19, 20].

Interestingly, this rhythmic process is essential for the regulation of many physiological functions, and its disruption is linked to numerous human diseases, including cancer [19, 21, 22]. In a study by Diamantopoulou et al. [23], circulating tumor cell (CTC) levels and their metastatic potential were assessed in women with early and metastatic breast cancer during sleep. The results indicated greater invasiveness of CTCs during the rest phase (4 am) compared to the active phase (10 am). This finding was also observed in rodent models, reflecting their circadian rhythms that are opposite those of humans. Furthermore, cancer cell‐transplanted *BMAL1* homozygous knockout mice showed reduced CTC release compared to their wild‐type counterparts, highlighting the significant impact of circadian rhythm disturbances on cancer progression.

Some studies have demonstrated that circadian genes influence cell death in normal and cancer cells. In 2011, Lee and Sancar [24] demonstrated that CRY expression induces apoptosis through NF‐κB signaling in dermal fibroblasts. Similarly, Qu et al. [25] reported that the downregulation of *BMAL1* and *CLOCK* increases apoptosis through p21 and WEE1 dysregulation in hepatocellular carcinoma cell lines. Moreover, circadian gene expressions such as *RORA, PER2, PER3*, and *CRY2* were correlated to cell cycle inhibition and apoptosis in renal cell carcinoma from animal models [26]. These findings suggest that circadian rhythm influences cell death in normal and cancer cells, which could be associated with fluctuations in total cfDNA and, consequently, with changes in ctDNA fraction and detectability.

Previously, Tóth et al. [27] investigated ctDNA levels and methylated Septin 9 (*SEPT9*) levels in colorectal cancer (CRC) patients. The Stage I‐III CRC patients predominantly showed cfDNA peaks at 6 am and 12 pm, and Stage IV patients peaked at midnight. Moreover, *SEPT9* methylation was detected at midnight in all CRC patients (9/9), and there was a correlation between the cfDNA level and *SEPT9* methylation in advanced stages (III and IV). However, Madsen et al. [28] and Poulet et al. [29] reported no significant circadian‐related variations in cfDNA levels among lung cancer patients and healthy subjects, respectively.

These studies were conducted in a small population, which prevents the generalization of the results obtained and their clinical application. Thus, these findings suggest that the circadian rhythm could influence cell death and cfDNA release, although the exact underlying mechanisms remain unknown. Therefore, more extensive cohort studies are needed to evaluate the impact of the circadian rhythm on ctDNA fraction and analytical sensitivity, as well as to determine whether standardized collection times could improve the reliability of ctDNA‐based assays.

## 3 Sex and Aging as Determinants of CFDNA Dynamics and Potential Implications for CTDNA Analysis

Biological sex significantly influences cfDNA levels due to differences in hormonal profiles and body composition. Males typically exhibit higher muscle mass, erythrocyte counts, and concentrations of androgens such as testosterone and dihydrotestosterone. In contrast, females generally present lower muscle mass and red blood cell counts alongside higher concentrations of estrogen hormones, including estradiol [30, 31]. However, the precise impact of these sex‐related differences on ctDNA fraction and analytical sensitivity in oncology remains largely unexplored [31, 32].

Aging, a complex biological process marked by the gradual decline of physiological functions and increased susceptibility to chronic diseases, is driven by numerous biological mechanisms [33]. During aging, oxidative stress escalates, and free radicals disrupt cell signaling pathways, leading to enhanced cell death [34, 35]. These changes significantly influence cfDNA dynamics, affecting its release, clearance, and molecular composition [36, 37]. Due to the limited availability of studies directly assessing the impact of biological sex and aging on ctDNA dynamics in cancer patients, the evidence discussed in this section is primarily derived from cfDNA studies and is presented to infer potential implications for ctDNA fraction and detectability.

Previous studies have demonstrated that cfDNA levels are significantly higher in older individuals compared to younger ones [38, 39]. For instance, Jylhävä et al. [38] reported significantly elevated cfDNA levels in plasma samples from nonagenarian women compared to younger control women (*p* < 0.05). Similarly, Zhong et al. [39] observed increased cfDNA levels in older patients relative to younger patients (*p* < 0.05), with a particularly notable increase in older females (*p* < 0.05). These findings underscore that aging enhances cell death, thereby increasing the release of cfDNA from tissues [37].

Recent research by Fisher et al. [40] revealed that postmenopausal women exhibit higher cfDNA levels compared to premenopausal women. However, the study did not conclusively determine whether this difference is attributable to hormonal changes or the aging process itself, due to the age disparity between the cohorts. Additionally, the majority of studies have predominantly focused on female populations, limiting the understanding of the effects of biological sex on cfDNA and ctDNA dynamics [29, 30, 31, 32, 33, 34, 35, 36, 37, 38, 39, 40].

Thus, further research is essential to elucidate the complex interplay between biological sex, aging, and cfDNA levels, as well as to determine whether these factors meaningfully influence ctDNA fraction and analytical performance in cancer patients. Specifically, studies incorporating male populations are needed to provide a more comprehensive understanding of these biomarkers and their clinical implications. Such efforts will enhance the utility of ctDNA as a diagnostic and prognostic tool across diverse demographic groups.

## 4 Can Diet‐Derived Metabolic Syndromes Affect the Analysis of ctDNA?

Dietary nutrients play a crucial role in maintaining homeostasis, and nutritional imbalances can contribute to the development of various metabolic syndromes [41]. Some dietary compounds, such as butyrate and fiber, influence epithelial cell turnover in the bowel, but their impact on cfDNA levels remains unclear [42]. Among diet‐related syndromes, obesity and diabetes mellitus are particularly associated with increased cancer risk and poorer survival outcomes in cancer patients [43, 44].

Excess adipose tissue promotes systemic inflammation by increasing proinflammatory cytokine levels, leading to enhanced cell death through necrosis and apoptosis, which in turn elevates cfDNA concentrations in the bloodstream [45]. A study investigating the relationship between diet and cfDNA levels in animal and human models found that obesity increases plasma levels of single‐stranded and double‐stranded DNA, as well as nucleosomes. These findings were correlated with increased visceral adipose tissue, inflammation, and insulin resistance, suggesting a link between cfDNA levels and obesity progression [46].

Similarly, Camuzi Zovico et al. [47] reported a positive correlation between body fat percentage and cfDNA levels (*r* = 0.310, *p* = 0.031) in 38 obese adults who underwent bariatric surgery. Additionally, studies in animal and human models have shown that obesity increases neutrophil levels and enhances NETosis, one of the primary sources of cfDNA [48, 49].

Diabetes mellitus is a diet‐derived condition that is characterized by glucose metabolism dysregulation and has several complications, including cardiovascular events, neuropathy, diabetic retinopathy, diabetic nephropathy, and others [50]. This condition has been linked to increased cell death in adipocytes and renal cells, but the direct impact of diabetes mellitus on plasma ctDNA levels remains unclear [51, 52, 53].

Collectively, these findings suggest that diet‐related chronic conditions associated with cancer may influence ctDNA levels and subsequent molecular analyses. Therefore, these factors should be carefully considered and accounted for in LB studies involving oncology patients.

## 5 Could Smoking Increase ctDNA Levels?

Smoking is associated with an increased incidence of various diseases, including cancer, and has a direct impact on the therapeutic response of oncology patients. Tobacco use negatively affects the efficacy of chemotherapy, radiotherapy, and immunotherapy, ultimately reducing survival rates and quality of life [54]. Additionally, smoking induces vascular smooth muscle cell death through ferroptosis. Thus, patients with such injuries exhibit higher cfDNA concentrations compared to control individuals, and cfDNA levels have been proposed as a biomarker of smoke inhalation injury severity. Moreover, elevated cfDNA levels have been correlated with both hospitalization and prolonged hospital stays [55, 56].

Studies in patients with nonsmall cell lung cancer (NSCLC) have shown that Epidermal Growth Factor Receptor (*EGFR*) mutations are more frequently detected in plasma samples from nonsmokers than from smokers. This difference may be due to increased cell death and subsequent cfDNA release associated with smoking [57, 58]. If the detection of *EGFR* mutations in NSCLC patients is akin to “looking for a needle in a haystack”, then smokers may be considered to have a larger haystack.

## 6 How Can Physical Exercise Affect ctDNA Levels?

The impact of physical exercise on the homeostasis of peripheral hematological cells is well documented, leading to increased cfDNA levels in plasma. In 2018, Hummel et al. [59] reported that physical stress contributes to cfDNA release, with peak levels increasing fivefold at 15 min after maximal physical exertion. Similarly, a recent study found that the intensity of physical exercise correlates directly with cfDNA concentration, which subsequently normalizes after approximately 1 h. This study also identified mature neutrophils as the primary source of cfDNA, with muscle cells and other cell types playing a minor role [60].

Moreover, evidence suggests that physical exercise enhances DNase activity, increasing the degradation of neutrophil extracellular traps (NETs) [61, 62]. Elevated cfDNA levels resulting from exercise or physical stress may act as a confounding factor in detecting clinically relevant ctDNA mutations [59, 60]. Therefore, this factor should be considered when providing recommendations to patients or collectors, allowing for logistical adjustments that ensure optimal sample collection conditions.

## 7 Should Tumor Staging and Therapeutic Approach Be Considered in ctDNA Analysis?

In oncology, ctDNA analysis has several applications as a source of diagnostic, prognostic, and predictive biomarkers, which may vary according to disease stage. In the early stages of cancer, ctDNA is mainly used to detect the disease. However, in advanced stages, it can be applied to monitor metastatic disease and select more effective therapies [7]. In general, ctDNA is more readily detectable in patients with metastatic disease than in patients with early‐stage disease and is associated with advanced disease in many cancer types, including ovarian, lung, and breast cancer [63, 64, 65].

Currently, several therapeutic modalities are available for cancer patients, such as chemotherapy, radiotherapy, targeted therapies, and immunotherapy. These treatment modalities aim to eliminate cancer cells or disable their growth and division. Cell death caused by these treatments temporarily increases ctDNA levels in the bloodstream [66, 67]. However, a decrease in ctDNA levels is observed if the treatment is effective once the tumor size and burden are proportional to the rate of ctDNA release [67]. This pattern is also observed in solid tumors treated with targeted therapies, such as melanoma and lung cancer [68, 69, 70].

Although most studies have shown a reduction in ctDNA levels in response to targeted therapies and immunotherapy, it is worth noting that the differences between the included methodologies (digital droplet polymerase chain reaction, targeted error correction sequencing, high‐throughput sequencing, and NGS) restrict the applicability of the results. Similarly, there was no consistency between the measurement intervals of ctDNA levels (which ranged from 6 to 16 weeks) after the beginning of treatment. However, across the cancer types evaluated, a significant reduction in ctDNA levels in response to treatment was associated with substantial improvements in outcome [68, 69, 70].

Surgery‐induced trauma significantly increases cfDNA release, which may impact the postoperative detection of ctDNA. Therefore, some studies focusing on the surgical impact that evaluate ctDNA levels are based on the difference between the variant allele frequency (VAF) in ctDNA and the mutation rate before and after surgery. Compared with preoperative patients, lung and colorectal cancer patients exhibit a decrease in mutations in ctDNA in postoperative plasma [71, 72].

Notably, the increase in cfDNA levels resulting from surgical trauma can persist for up to 4 weeks, potentially leading to a ctDNA fraction that falls below the assay detection limit. Furthermore, ctDNA levels are anticipated to be low following surgery, particularly with minimal tumor burden and collection occurring after 5 weeks. Thus, methods with low sensitivity (such as variant allele frequency [VAF] of 0.06%) are more likely to detect ctDNA during Week 5 than in the first week postsurgery [73]. According to ESMO recommendations regarding the use of ctDNA assays, it is advisable to collect blood at least 1–2 weeks after the surgical procedure, depending on the extent of tissue injury and the duration of recovery [74].

## 8 Impact of Collection Tubes and Processing Time on ctDNA Analysis

ctDNA analysis in blood can be performed using either serum or plasma; however, plasma is preferred. Plasma samples are collected in tubes containing anticoagulants, which prevent leukocyte lysis during centrifugation. In contrast, leukocyte lysis in serum leads to a substantial release of genomic DNA, potentially diluting the tumor‐derived DNA fraction and compromising the ctDNA molecular analysis [75].

Among the various anticoagulants, K2‐ and K3‐EDTA are particularly suitable for ctDNA analysis due to their ability to inhibit DNase activity, protect cells from degradation, and not interfere with PCR‐based processes. However, within 4–6 h after blood collection in EDTA tubes, the total DNA concentration tends to increase as a result of leukocyte lysis [32, 76, 77]. This process results in an increase in wild‐type DNA (nonneoplastic), which can obscure the presence of ctDNA and lead to false‐negative results and misinterpretations [78, 79].

Several studies have compared the use of specialized blood collection tubes that are designed to enhance the preservation of ctDNA by prolonging its half‐life. These tubes incorporate cell‐stabilizing additives that mitigate enzymatic degradation and cellular lysis, thereby maintaining ctDNA integrity for a longer period before processing. As a result, they allow for extended time intervals between sample collection and downstream analysis without significant loss of nucleic acid quality. This improved stability has important implications for the reliability of LB assays, particularly in clinical settings where immediate processing is not feasible [15, 78, 80, 81, 82].

Despite the advantages provided by tubes containing specialized additives, which have been shown to prolong the half‐life and enhance the preservation of ctDNA, EDTA tubes continue to be the preferred choice in the majority of studies. This preference is primarily due to their low cost and their entrenched use in routine hospital workflows. Thus, the marginal benefits of extended ctDNA stability do not outweigh the economic and logistical advantages of EDTA tubes. Consequently, EDTA tubes remain the standard for ctDNA collection and analysis in both clinical and research environments.

## 9 Comparison of cfDNA Isolation Methods and Storage Considerations

The extraction of cfDNA from plasma is a critical step in LB analysis, directly influencing the sensitivity and specificity of genetic assessments [83, 84]. Various commercial methods have been evaluated in comparative studies, with column‐ and magnetic bead‐based approaches standing out (Table 1).

**TABLE 1:** Isolation techniques used in ctDNA analysis.

Authors	Isolation kit/Method	Operation	Technique	Conclusion
Sorber et al. [84]	QIAamp Circulating Nucleic Acid Kit	Manual	Silica columns	QIAamp/Maxwell had better yield
	PME free‐circulating DNA Extraction Kit	Manual	Interaction with polymer	
	Maxwell RSC LV ccfDNA Plasma Custom Kit	Automatized	Magnetic beads	
	EpiQuick Circulating Cell‐Free DNA Isolation Kit	Manual	Silica columns	
	NEXTprep‐Mag cfDNA Isolation Kit (v1)	Manual	Magnetic beads	
	NEXTprep‐Mag cfDNA Isolation Kit (v2)	Automatized	Magnetic beads	
Van Dessel et al. [85]	QIAamp Circulating Nucleic Acid Kit	Manual	Vacuum columns	QIAamp/QIAsymphony had better yield
	QIAsymphony SP Circulating DNA Kit	Automatized	Magnetic beads	
	Maxwell RSC LV ccfDNA Plasma Custom Kit	Automatized	Magnetic beads	
Sherwood et al. [83]	QIAamp Circulating Nucleic Acid Kit	Manual	Silica columns	QIAamp had better yield
	PME free‐circulating DNA Extraction Kit	Manual	Magnetic beads	
	QIAsymphony DSP Virus/Pathogen Midi Kit	Automatized	Magnetic beads	
Kresse et al. [86]	QIAamp Circulating Nucleic Acid Kit	Manual	Silica columns	QIAamp had better yield
	Maxwell RSC LV ccfDNA Plasma Custom Kit	Automatized	Magnetic beads	
	QIAamp MinElute ccfDNA Mini Kit	Manual/automatized	Silica columns	
Wang et al. [87]	QIAamp Circulating Nucleic Acid Kit	Manual	Silica columns	QIAamp had better yield
	AmoyDx Circulating DNA kit	Manual	Silica columns	
	Microdiag circulating DNA isolation kit	Manual	Silica columns	
	MagMAX cell‐free DNA isolation kit	Manual/automatized	Magnetic beads	
Pérez‐Barrios et al. [88]	QIAamp Circulating Nucleic Acid Kit	Manual	Silica columns	QIAamp/Maxwell had better yield
	MagNA Pure Compact Nucleic Acid Isolation Kit I	Automatized	Magnetic silica columns	
	Maxwell RSC LV ccfDNA Plasma Custom Kit	Automatized	Magnetic beads	
Kerachian et al. [89]	Selective capture of ctDNA on magnetic beads	Experimental	Functionalized microspheres	Satisfactory yield
Pandoh et al. [90]	Isolation method of cfDNA directly from peripheral blood	Experimental	Magnetic beads	Satisfactory yield
Varona et al. [91]	Solid‐phase microextraction technique with a polymeric ionic liquid coating	Experimental	Solid‐phase microextraction	Satisfactory yield
Lee et al. [92]	Specific Gravity‐Free Method	Experimental	Dual isolation system	Satisfactory yield
Krug et al. [93]	Exolution Plus	Manual/automatized	Spin columns	Satisfactory yield

Among widely used methods, column‐based protocols have demonstrated high efficiency in cfDNA recovery, particularly in samples with low tumor burden. Studies indicate that this approach offers greater sensitivity in detecting low‐frequency mutations, making it a reliable choice for mutational status analysis [83, 84, 85, 86, 87]. On the other hand, magnetic bead‐based methodologies have been developed as viable alternatives, showing efficiency comparable to column‐based methods. Additionally, some of these techniques offer the advantage of automation, facilitating large‐scale processing and reducing variability introduced by manual procedures [84, 88].

However, other methodologies, including those combining polymers and magnetic beads, have shown lower yields and the presence of inhibitors in the elution solution, potentially compromising digital PCR amplification. These limitations restrict the applicability of such techniques for highly sensitive molecular analyses [84].

Beyond commercial methods, experimental approaches have been investigated, including modified magnetic bead‐based extraction [89, 90], solid‐phase microextraction [91], and co‐extraction of cfDNA and exosomes [92, 93]. While some of these strategies have demonstrated good yields, the lack of standardized comparative studies limits the assessment of their effectiveness relative to established methodologies.

After extraction, proper plasma storage is essential for preserving cfDNA integrity. Evidence suggests that temperatures of −80°C are ideal for maintaining cfDNA stability for up to 9 months [94, 95]. However, long‐term storage can lead to gradual DNA degradation, which should be considered when planning retrospective analyses [96]. Given these considerations, the choice of extraction method should account not only for yield and analytical sensitivity but also for scalability and operational feasibility across different applications.

## 10 Conclusions and Future Perspectives

LB has become an essential tool in precision medicine, and understanding the preanalytical factors required to collect and process samples adequately is crucial for obtaining reliable results. Despite some ctDNA‐based tests being used in many clinical trials and some approved for use in clinical management by the FDA for metastatic melanoma, prostate, lung, colorectal, and breast cancer, there is still no standard of preanalytical indications for this technique [7, 97].

**FIGURE 2:**
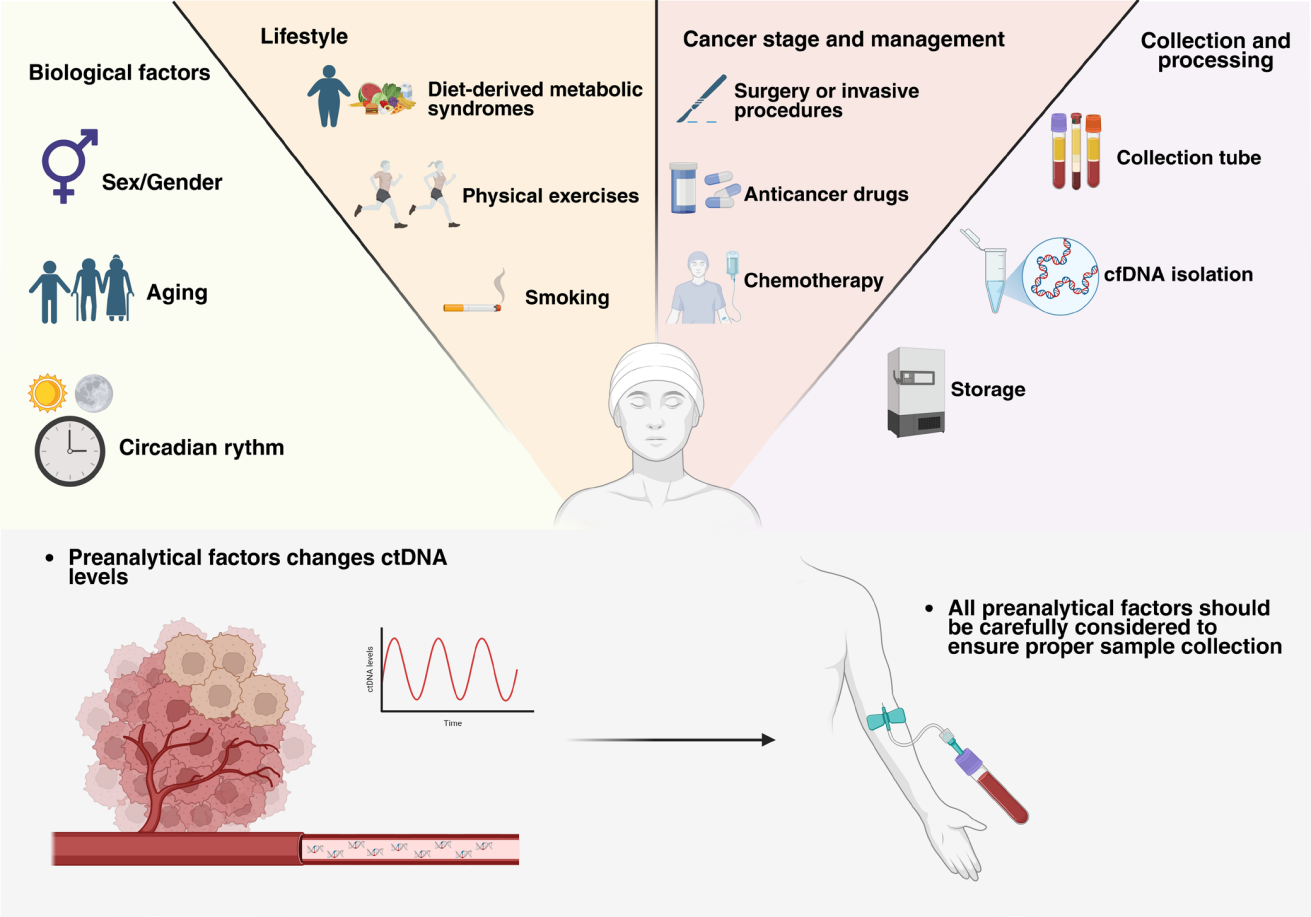
Overview of preanalytical factors affecting ctDNA analysis, including biological characteristics, lifestyle influences, cancer stage and treatment, as well as sample collection and processing considerations.

Since 2018, the American Society of Clinical Oncology (ASCO) and the College of American Pathologists (CAP) have emphasized the critical impact of preanalytical variables on the reliability of ctDNA testing [97]. This emphasis is necessitated by the fact that total cfDNA levels are highly dynamic, influenced by a multifaceted array of factors—ranging from biological and lifestyle aspects to cancer staging, treatment regimens, and specific collection and processing techniques (Table 2, Figure 2).

**TABLE 2:** Summary of preanalytical factors that influence cfDNA levels and ctDNA analysis.

Preanalytical factor	Influence	References
*Biological*		
Circadian rhythm	Circadian genes influence cell death in normal and cancer cells, elevating cfDNA levels during the rest phase	[24, 25, 26, 27]
Sex	Men present higher levels of cfDNA compared to women	[31, 32]
Aging	Aging increases cell death and cfDNA levels	[38, 39, 40]
*Lifestyle*		
Diet‐derived syndromes metabolic	Obesity and DM induce systemic inflammation and cell death and increase cfDNA levels in the bloodstream	[47, 48, 49]
Smoking	Smoking increases cell death and cfDNA levels	[55, 56, 58]
Physical exercises	Temporarily elevates cfDNA levels and increases DNase activity	[59, 60, 61, 62]
*Treatment*		
Therapies	Chemo‐, radiotherapy, and targeted therapies temporarily increase ctDNA levels	[65, 66]
Surgery	Surgery increases cfDNA and impact on postoperative ctDNA detection	[70, 71, 72]
*Sample collection*		
Collection tubes	EDTA tubes induce leukocyte cell death and increase cfDNA levels. BCT has higher stability	[75, 79]
Isolation of ctDNA	Commercial ctDNA isolation kits have shown efficiency in isolating ctDNA from plasma, even in samples with low variant allele frequency	[83, 85, 86]

Although ctDNA is part of the cfDNA pool, it represents only a small fraction of total circulating DNA. Most cfDNA originates from nonneoplastic tissues, particularly hematopoietic cells. Therefore, preanalytical and biological factors may differentially affect total cfDNA concentration and the relative fraction of ctDNA. Importantly, several conditions increase cfDNA levels without increasing ctDNA, leading to dilution of tumor‐derived DNA and reduced assay sensitivity. Increased cfDNA derived from these preanalytical factors could camouflage ctDNA and provide false‐negative results. Thus, the real impact of these factors on the molecular analysis of LB in oncology patients needs to be investigated in subsequent studies with a larger cohort.

In 2022, the FDA made available draft guidance for the industry entitled “Use of Circulating Tumor DNA for Early‐Stage Solid Tumor Drug Development”, which helps further develop the use of ctDNA as a biomarker in cancer clinical trials for new experimental drug applications and supports the approval of drugs for the treatment of early‐stage solid tumors [98]. Overall, this review suggests that various physiological and external factors—such as physical exercise, tumor burden, therapeutic approach, and smoking—can significantly influence cfDNA and ctDNA levels, potentially affecting their detection and clinical interpretation. Therefore, these variables should be carefully considered in preanalytical protocols to ensure the reliability and accuracy of LB analyses.

## Author Contributions

**Juscelino Carvalho de Azevedo Junior:** conceptualization, data collection, critical review and analysis, visualization, writing – original draft and editing. **Douglas Rafael da Cruz Carneiro:** data collection, critical review and analysis, visualization, writing – original draft. **Fernanda Jardim da Silva:** critical review and analysis, visualization, writing – original draft and editing. **Ana Beatriz Lima Belicha and Anna Carolina Lima Rodrigues:** data collection and writing – original draft. **Williams Fernandes Barra and Alessandro Leal:** critical review and analysis, visualization, writing – original draft and editing. **Danielle Queiroz Calcagno:** conceptualization, supervision, data collection, critical review and analysis, visualization, writing – original draft and editing. All authors contributed to the article and approved the submitted version.

## Funding

This work was financially supported by the Universidade Federal do Pará, Ministério da Educação, Coordenação de Aperfeiçoamento de Pessoal de Nível Superior, and Conselho Nacional de Desenvolvimento Científico e Tecnológico (CNPq; to Danielle Q. Calcagno, #409332/2021‐6 and #315643/2023‐4). The Article Processing Charge for the publication of this research was funded by the Coordenação de Aperfeiçoamento de Pessoal de Nível Superior—Brasil (CAPES) (ROR identifier: 00x0ma614).

## Ethics Statement

The authors have nothing to report.

## Consent

The authors have nothing to report.

## Conflicts of Interest

The authors declare no conflicts of interest.

## Data Availability

Data sharing not applicable to this article as no datasets were generated or analyzed during the current study.
